# Predicting El Niño in 2014 and 2015

**DOI:** 10.1038/s41598-018-29130-1

**Published:** 2018-07-16

**Authors:** Sarah Ineson, Magdalena A. Balmaseda, Michael K. Davey, Damien Decremer, Nick J. Dunstone, Margaret Gordon, Hong-Li Ren, Adam A. Scaife, Antje Weisheimer

**Affiliations:** 1 Met Office Hadley Centre, Exeter, UK; 2 European Centre for Medium- Range Weather Forecasts, Reading, Berks UK; 3 Department of Applied Mathematics and Theoretical Physics, University of Cambridge, Cambridge, UK; 4 Laboratory for Climate Studies & CMA-NJU Joint Laboratory for Climate Prediction Studies, National Climate Center, China Meteorological Administration, Beijing, 100081 China; 5 Department of Atmospheric Science, School of Environmental Studies, China University of Geoscience, Wuhan, 430074 China; 6 College of Engineering, Mathematics and Physical Sciences, University of Exeter, Exeter, UK; 7 National Centre for Atmospheric Science (NCAS), Department of Atmospheric, Oceanic and Planetary Physics, University of Oxford, Oxford, UK

## Abstract

Early in 2014 several forecast systems were suggesting a strong 1997/98-like El Niño event for the following northern hemisphere winter 2014/15. However the eventual outcome was a modest warming. In contrast, winter 2015/16 saw one of the strongest El Niño events on record. Here we assess the ability of two operational seasonal prediction systems to forecast these events, using the forecast ensembles to try to understand the reasons underlying the very different development and outcomes for these two years. We test three hypotheses. First we find that the continuation of neutral ENSO conditions in 2014 is associated with the maintenance of the observed cold southeast Pacific sea surface temperature anomaly; secondly that, in our forecasts at least, warm west equatorial Pacific sea surface temperature anomalies do not appear to hinder El Niño development; and finally that stronger westerly wind burst activity in 2015 compared to 2014 is a key difference between the two years. Interestingly, in these years at least, this interannual variability in wind burst activity is predictable. ECMWF System 4 tends to produce more westerly wind bursts than Met Office GloSea5 and this likely contributes to the larger SST anomalies predicted in this model in both years.

## Introduction

A series of westerly wind bursts (WWBs) from January to April 2014 increased the already positive heat content anomaly in the upper tropical Pacific Ocean and also increased the sea surface temperature (SST) in the central/east equatorial Pacific. Similarities between this and the ocean state prior to the 1997/98 El Niño event led to anticipation of a similar strong event for 2014/15 (ref.^1^). However, large scale coupling (mutual amplification of SST and wind anomalies) between the ocean and atmosphere failed to occur and warming was only modest, so that in winter 2014/15, SST was close to weak El Niño levels and some atmosphere indicators (in particular, cloudiness and rainfall patterns) remained neutral^2^. Conversely, WWBs and comparably high ocean heat content in 2015 were accompanied by large scale development with the result that the 2015/16 event was one of the strongest El Niños in the recent observational record^3^.

The effects of El Niño Southern Oscillation (ENSO) are felt worldwide (e.g. ref.^4^) and societal impacts can be significant, especially so for the strongest El Niño events such as 1982/83 and 1997/98 (ref.^5^). Hence understanding the very different observed trajectories of the tropical Pacific state between 2014 and 2015 is of great importance. Dynamical forecast systems are expected to give the most accurate forecasts but challenges still remain both in forecast skill and reliability, and in making best use of ensemble information. To this end, understanding the differences between these two years, the differences between the forecast systems, and why a large event failed to materialise in 2014/15, are key questions.

A similarity between 2014 and 2015 is that in both years, SSTs were warmer than normal in the north Pacific, with a Pacific Meridional Mode (PMM)-like SST anomaly pattern^6^ that could raise the likelihood of El Niño development^7,8^. However, in 2014 there was also a persistent region of negative SST anomalies south of the equator in the southeast Pacific. A number of studies suggest that the anomalous meridional SST gradient between the northeast and southeast Pacific in 2014 may have led to the suppression of ocean-atmosphere interaction^9–12^.

By late spring 2014 SSTs in the east equatorial Pacific had warmed due to the arrival of Kelvin waves associated with the earlier WWBs but other ENSO indicators remained neutral indicating that the atmosphere was failing to engage with the ocean to amplify anomalies. A possible reason for this was that above average SSTs extended into the western tropical Pacific, so that the strong anomalous west to east SST gradients required for ocean-atmosphere feedback had not yet been established^13^, limiting the development of westerlies in the central equatorial Pacific^14^.

A hiatus in WWBs followed the late winter and early spring activity in 2014 with WWBs being absent in May and June. The lack of events in 2014 is thought to have limited the potential for El Niño growth in 2014 (refs^14,15^). In contrast in 2015 there was activity throughout the year and the role of WWBs in the development of the strong 2015/16 event has been highlighted^16,17^. Rather than the absence of WWBs in 2014, an alternative hypothesis is that strong easterly bursts in June and July stalled development^18^, and indeed potentially sowed the seeds for the large event in 2015 (ref.^19^). Simulation of the observed Pacific SST variability in 2014 and 2015 with an ocean model forced by reconstructed wind stress fields demonstrates the importance of westerly and easterly subseasonal wind variability in these years^20^.

During 2014 evidence emerged that the slowly varying basin scale pattern of Pacific Ocean variability, which had been in a mostly negative state over the past decade, might be moving to a more persistently positive state^21^. Using the POAMA seasonal forecast system, it has been demonstrated that changes to the equatorial ocean mean state affected El Niño development during 2014 and 2015 (ref.^22^), with a cold (warm) Interdecadal Pacific Oscillation state acting to reduce (enhance) coupled feedbacks and reduce (enhance) predictability^23^. The ocean components of the forecast systems used here are initialised with the full observational state and therefore will, in principle, capture these differences.

In this study we show the results from forecasts initialised in May 2014 and May 2015 from the Met Office GloSea5 system and ECMWF System 4 (G5 and S4 hereafter, see Methods). We then consider three hypotheses motivated by the above literature: that the region of cold SST in the southeast Pacific in 2014 played a role in the suppressed El Niño development in that year, that the warm SST anomaly in the west Pacific in 2014 hindered the development of El Niño, and that differences in WWBs between the two years explains the different ENSO evolution.

## Results

### Forecast plumes

**Figure 1:**
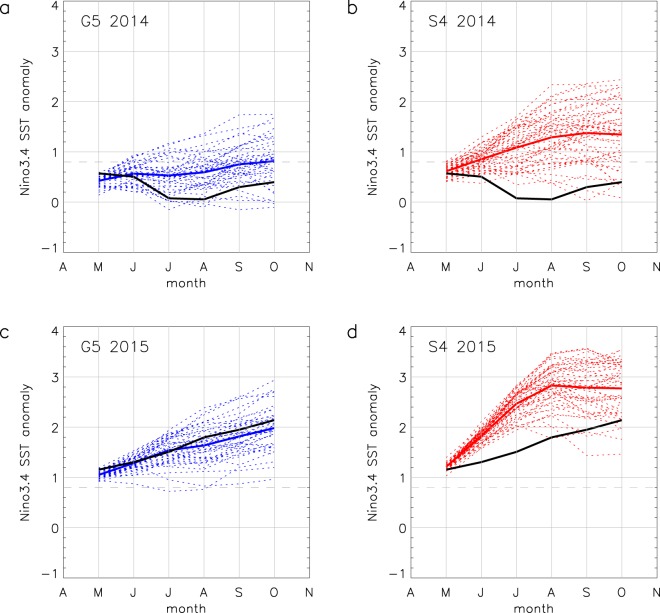
Plume diagrams for May 2014 and May 2015 forecasts. May to October forecast ensemble members (dashed lines) for G5 (42 members, blue) and S4 (51 members, red) for region Niño3.4 (170°W-120°W, 5°N-5°S) for (**a**) G5 2014 (**b**) S4 2014 (**c**) G5 2015 and (**d**) S4 2015. Solid coloured lines show the ensemble means and observations (black line) are HadISST1.1. Anomalies for both forecasts and observations are calculated with respect to a 1996–2009 climatology (see Methods). The dashed grey line indicates the 0.8 °C threshold.

In May and June 2014 the observed monthly mean SST anomaly in region Niño3.4 (170°W-120°W, 5°N-5°S) had risen from slightly below zero early in the year to nearly 0.6 °C. It then decreased and was near zero during July and August before slowly increasing in September and October to approach 0.4 °C. The ensemble means for both G5 (Fig. 1a) and S4 (Fig. 1b) forecasts are greater than observations throughout the forecast, and both show a steady increase in SST anomaly with October means of 0.8 °C and 1.3 °C respectively, neither capturing the observed cooler July-August period. For each month, the G5 ensemble mean is closer to the observations than S4, although we would not necessarily expect the ensemble mean to match observations. Considering the behaviour of ensemble members, we see that for July and August all members of the S4 ensemble and most of G5 are warmer than the observations. However, for G5, some individual ensemble members cool in July relative to the initial conditions, such that the observations lie within the G5 ensemble plume.

By May 2015, observed SSTs had warmed somewhat in the central and eastern equatorial Pacific relative to 2014 and the Niño3.4 SST anomaly was just above 1.1 °C. During the following months there was a steady warming with the Niño3.4 SST anomaly exceeding 2.1 °C in October. From May 2015, both G5 (Fig. 1c) and S4 (Fig. 1d) indicate warming, with October ensemble mean Niño3.4 SST anomalies of 2.0 °C and 2.8 °C respectively. The G5 ensemble mean agrees well with the observations throughout the forecast. During May-June-July S4 shows an exaggerated rate of warming, so that the forecast plume is warmer than the observations. During the second half of the forecast the warming levels off.

The 6-month lead spread of these ensembles (calculated as standard deviation) averaged over 2014 and 2015 is larger for S4 (0.56) than for G5 (0.44). This also applies to the April and June initialised forecasts.

We now use the range of behaviours in these ensemble predictions to test a series of hypotheses for the development of the strong El Niño in 2015 but not in 2014.

### Hypothesis 1: Did the cold southeast Pacific SST anomaly hinder El Niño development in 2014?

**Figure 2:**
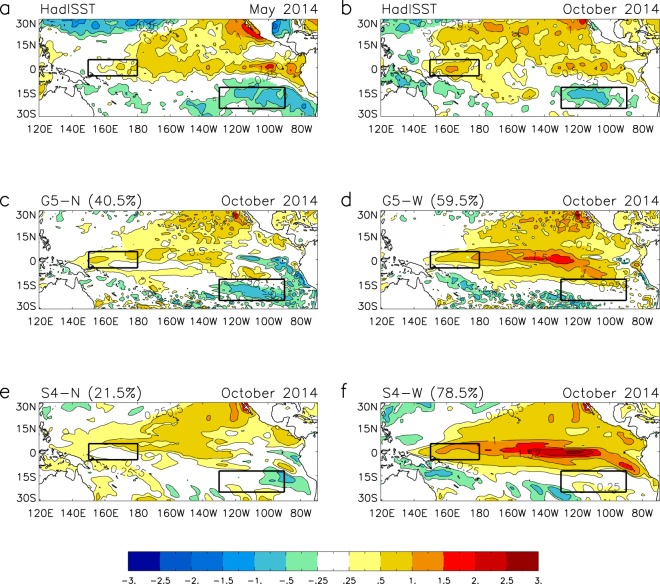
Categorising forecast ensemble members initialised in May 2014 as ‘neutral’ or ‘warm’ ENSO composites. Observed (HadISST1.1) tropical Pacific SST anomaly for (**a**) May 2014 and (**b**) October 2014. Composite forecast SST anomalies for October 2014 for (**c**) ‘neutral’ G5 members, (**d**) ‘warm’ G5 members, (**e**) ‘neutral’ S4 members and (**f**) ‘warm’ S4 members. Categories for composites are based on forecast October SST anomaly: −0.8 °C < ‘neutral’ < 0.8 °C ≤ ‘warm’. Boxes show the west Pacific area (150°E-180°, 5°N-5°S) and the southeast Pacific area (130°W-90°W, 12°S-25°S). Figure was created using IDL8.2 (http://www.harrisgeospatial.com/SoftwareTechnology/IDL.aspx), Met Office (in-house) release 8.2.0.10.

SST observations for May 2014 (Fig. 2a) show warm equatorial anomalies at all longitudes except the far west Pacific. Tropical warm anomalies are also present in the northeast Pacific and a region of lower than normal SST is present in the southeast Pacific. In October 2014 (Fig. 2b) equatorial anomalies, while still generally warm, have cooled somewhat in the central-east Pacific region, and warmed to the west of the dateline. Warming in the northeast Pacific remains, and the cold southeast Pacific anomaly, which had become established during the winter of 2013/14, is also still present. This cold anomaly persisted throughout 2014, finally dissipating in spring 2015.

To examine the intra-ensemble behaviour we have split the G5 and S4 ensemble forecasts from May 2014 into ‘warm’ and ‘neutral’ composites based on the forecast values in October. For this purpose, the warm composite includes members for which the October Niño3.4 anomaly is greater than 0.8 °C, and the neutral composite includes members with anomalies between −0.8 °C and 0.8 °C (0.8 °C is close to one standard deviation for observed Niño3.4 SST anomalies, but the results are similar for other thresholds, e.g. 0.5 °C). As might be expected from Fig. 1, more neutral members occur in the G5 ensemble than in S4, with 41% of G5 members and 22% of S4 members falling into this category.

October SST anomalies for the ‘correct’ neutral forecast composites (Fig. 2c and e) show very similar off equatorial anomalies to the observations (Fig. 2b). The warming in the northeast Pacific is still present, and most strikingly, the cold southeast Pacific anomaly has been maintained. This latter feature may also explain some of the differences between G5 and S4 as it is clearer in the G5 composite, whereas in S4 the there has been erosion from the western side, consistent with the generally higher ENSO forecasts in S4.

The key feature of the October warm composites (Fig. 2d and f) is that the cold southeast Pacific anomaly is absent, consistent with an influence of southeast Pacific SST on ENSO development. Warm northeast Pacific anomalies are still evident and the developing El Niño has largest anomalies in region Niño3.4 suggesting that the developing El Niño in these composites has mixed Central-Pacific (CP) and East Pacific (EP) El Niño characteristics. This would be consistent with the warm PMM-like pattern^24^ and a similarity with the 2015/16 event^25^^.^

**Figure 3:**
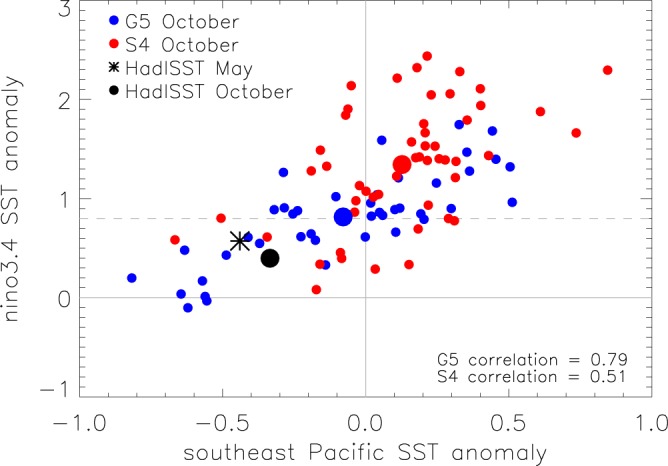
Relationship between El Niño and southeast Pacific SST for forecasts initialised May 2014. Scatter diagram of forecast ensemble members showing October 2014 Niño3.4 anomaly against October 2014 southeast Pacific SST anomaly for G5 (blue circles) and S4 (red circles). Ensemble means are large circles. Observations for May 2014 (black star) and October 2014 (black circle) are HadISST1.1. The dashed grey line indicates the 0.8 °C threshold. The southeast Pacific area (130°W-90°W, 12°S-25°S) is shown in Fig. 2.

We further explore the link between ENSO development and the southeast Pacific region in the forecasts by showing the relationship between the Niño3.4 SST anomaly and the SST anomaly for the southeast Pacific region (130°W-90°W, 12°S-25°S) for October 2014 for individual ensemble members (Fig. 3). Both G5 and S4 show a similar relationship, with higher forecast ENSO anomalies tending to be associated with higher SST anomalies in the southeast Pacific region, the correlation for the combined set is 0.67. Observations in the southeast Pacific region show little change in SST anomaly throughout the forecast period. While both forecast systems have some members which remain cold in the southeast Pacific, G5 appears better able to maintain these anomalies than S4. Hence the lack of cold southeast Pacific SST anomaly potentially explains both the difference between the observations and the forecasts, and the difference between the two forecast systems.

**Figure 4:**
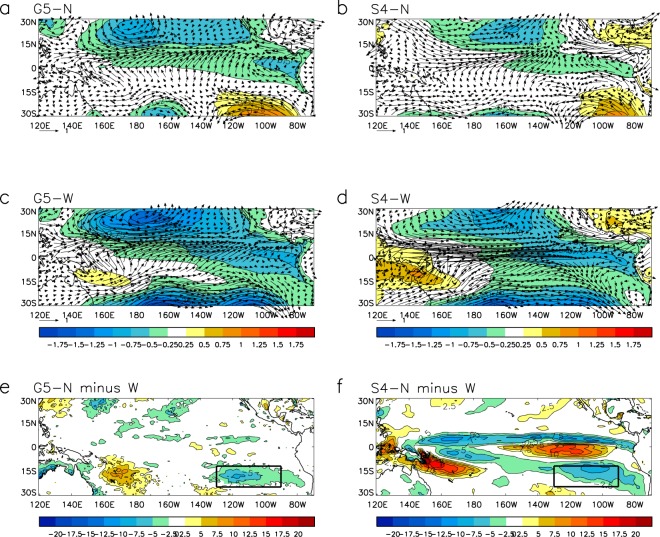
WES mechanism active in 2014? Mean May to October sea level pressure anomalies (colour) and 850hPa wind anomalies (wind arrows) for forecasts initialised in May 2014 for (**a**) ‘neutral’ G5 members, (**b**) ‘neutral’ S4 members, (**c**) ‘warm’ G5 members, (**d**) ‘warm’ S4 members, and difference in latent heat flux (downward positive) for ‘neutral’ minus ‘warm’ cases for (**e**) G5 and (**f**) S4. Categories for composites are as defined in Fig. 2. Boxes show the southeast Pacific area (130°W-90°W, 12°S-25°S). Figure was created using IDL8.2 (http://www.harrisgeospatial.com/SoftwareTechnology/IDL.aspx), Met Office (in-house) release 8.2.0.10.

Several observational and modelling studies have indicated that the process of wind-evaporation-SST (WES) feedback^26^ may have been active and played a part in suppressing El Niño development in 2014 (e.g. refs^9–12^). Associated with the forecast east Pacific meridional SST gradient in the neutral composites (Fig. 2c and e), we see that for both G5 and S4 anomalously high sea level pressure is maintained in the southeast Pacific and anomalously low pressure to the north of the equator throughout the forecast period, leading to a distinct south to north pressure gradient in the east Pacific (Fig. 4a,b). The southeasterly trade winds, cross equatorial flow and convergence into the ITCZ north of the equator are enhanced relative to climatology and overall there is an increased easterly zonal wind component in the equatorial east Pacific which will tend to suppress El Niño. In contrast the warm composites show anomalously low pressure both to the south and north of the equator in the central and east Pacific, decreased southeasterly trade winds and enhanced equatorial westerlies as expected during El Niño development (Fig. 4c,d). The difference in latent heat flux between the neutral and warm composites indicates that heat loss from the ocean in the region of the southeast Pacific cold anomaly is greater for the neutral composites (Fig. 4e,f). This suggests consistency with WES, with the anomalous SSTs being maintained through increased evaporation associated with the enhanced southeasterlies.

### Hypothesis 2: Could development of El Niño have been hampered by the warm equatorial west Pacific SSTs in spring 2014?

**Figure 5:**
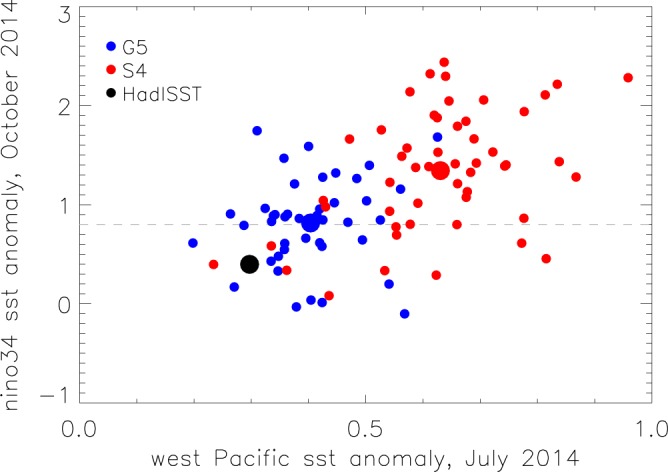
Relationship between El Niño and west Pacific SST for forecasts initialised May 2014. Scatter diagram of forecast ensemble members showing October Nino3.4 SST anomaly against July west Pacific SST anomaly for G5 (blue circles) and S4 (red circles). Ensemble means are large circles. Observations (black circle) are HadISST1.1. The dashed grey line indicates the 0.8 °C threshold. The west Pacific area (150°E-180°, 5°N-5°S) is shown in Fig. 2.

Figure 5 is a scatter plot of October 2014 Niño3.4 against the July SST anomaly in the west Pacific. If this hypothesis was supported by the forecast systems then a negative correlation would be expected, that is, a less warm west Pacific SST would encourage coupling between the ocean and atmosphere and hence El Niño development. However, this relationship is not observed in the ensemble members, as there is no significant relationship in G5, and a *positive* correlation (0.44) in S4, that is, El Niño development is associated with *increased* warming in the west Pacific. Correlation for the combined forecasts is 0.53. On the basis of our forecast ensembles this hypothesis is rejected.

### Hypothesis 3: Was the different El Niño development in 2014 and 2015 associated with the difference in WWBs?

Here we examine the WWBs simulated by the forecast systems in each year to determine whether the accumulated strength of forecast wind bursts was different in the two years and also to determine if the wind bursts in this period were related to the forecast October ENSO state. As numbers of WWBs are observed to be dependent on ENSO state^27^, we focus on WWBs in the first two months after initialisation (May-June), before forecast SST in individual ensemble members has diverged too far from the initial state.

**Figure 6:**
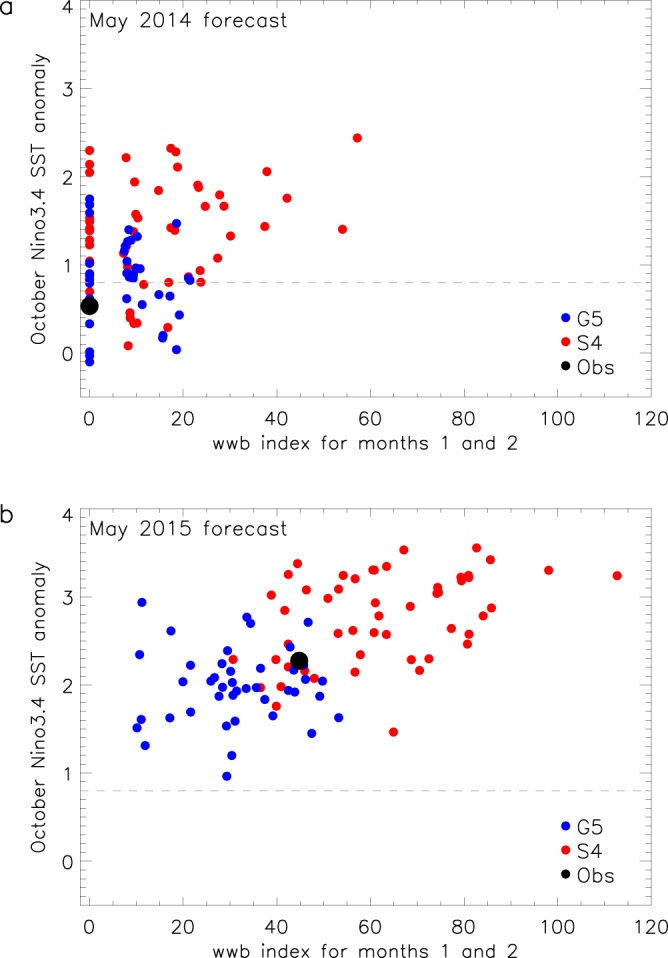
The impact of westerly wind bursts on El Niño development in May 2014 and May 2015 forecasts. Scatter diagram of forecast ensemble members showing October Niño 3.4 SST anomaly against a westerly wind burst index (see Methods) made for the first two months of the forecast for G5 (blue circles) and S4 (red circles). Observations (black circles) are HadISST1.1 and ERA-Interim (u10 winds). The dashed grey line indicates the 0.8 °C threshold.

Our WWB index (WWBI, see Methods) for the first two months of the May 2014 forecasts (Fig. 6a) is zero for nearly 30% of combined ensemble members (17/42 in G5 and 9/51 in S4) showing that these produce no WWBs. These members agree with observations, as no WWBs occurred in May or June. While G5 shows only small values of WWBI and hence little apparent relationship with El Niño development, for S4 there are larger WWBIs in some members and there is a suggestion that ENSO-neutral conditions are less likely to occur if the WWBI is high. However, for both G5 and S4 WWBI in the first two months is clearly not the only factor determining ENSO development as some forecast ensemble members fall into the El Niño category even with low or zero WWBI. The mean WWBI is considerably greater for S4 (14.6 ms^−1^) than G5 (7.2 ms^−1^) which also helps to explain why more S4 forecast members developed El Niño in 2014.

For May 2015 forecasts, the average WWBI is greater than for the May 2014 forecasts and no ensemble members produce an index of zero (Fig. 6b). There now also appears to be a clearer relationship between wind burst index and final forecast ENSO temperature anomaly, with a combined G5 and S4 correlation of 0.53 (G5 = 0.1, S4 = 0.43). The WWBI is generally higher for S4 than G5, with a mean WWBI of 31.5 ms^−1^ for G5 and 62.6 ms^−1^ for S4, with the observed index lying at the upper end of the G5 distribution and toward the lower end of S4.

Our definition of WWBI analyses the zonal wind anomaly, which encompasses variability on a range of timescales, including relatively short-lived westerly wind bursts and the interannual wind variability associated with ENSO. To address whether removing the low frequency component makes a difference to our results we have diagnosed the linear response of the mean low-level wind to east Pacific SST (Supplementary Fig. S1) and recalculated the WWBI after removing this component for each forecast member (Supplementary Fig. S2). As expected, this reduces the westerly wind burst count and WWBI where SST is large, however the key results described above continue to hold.

Overall, these results are consistent with the hypothesis that increased WWB activity in 2015 relative to 2014 played a role in the difference between forecast ENSO behaviour in spring 2014 and spring 2015.

## Discussion

Using seasonal forecast ensembles for G5 and S4 we have tested three hypotheses for the very different ENSO evolution in 2014 and 2015.

We find that both ensembles support the theory that the cold southeast Pacific SST anomaly was associated with lack of El Niño development in 2014. Off equatorial SST anomalies in the South Pacific (the South Pacific Meridional Mode) have been shown to have the potential to influence ENSO development^24,28^. The impact of the lack of prediction of the cold southeast Pacific anomaly in 2014 has also been explored in the NCEP CFSv2 seasonal prediction system and has been found to explain 40% of the positive peak amplitude error in SST anomaly for Niño3.4 (ref.^11^). The mechanism for maintaining the anomalous north-south SST gradient is identified as WES feedback^26^ with a possible role for stratus-SST feedback^24^ and these processes are thought to be poorly represented by some models^11,29^. However, we have shown here that both G5 and S4 forecast ensembles have some model solutions capable of maintaining the southeast Pacific anomaly, especially in G5, and that the mechanism appears to be consistent with WES feedback.

Neither S4 nor G5 supported the hypothesis that warm west Pacific SSTs hampered subsequent El Niño growth in 2014 with the combined forecasts showing an overall positive (rather than negative) relationship. However, there are a few caveats to this conclusion. We note that a positive anomaly in the equatorial west Pacific is (correctly) maintained in 2014 in most ensemble members which somewhat limits the exploration of this hypothesis. In addition, the forecast relationship in S4 could be partially due to model error, as the El Niño SST signal extends unrealistically far into the west Pacific (Fig. 2f). Examination of the large scale Pacific SST gradient (not shown) indicates a close relationship in the ensemble between the anomalous west to east SST gradient and future El Niño growth consistent with ocean-atmosphere feedback^30^, and that by July, the majority of ensemble members had developed a stronger anomalous gradient than observed, despite their warmer west Pacific SST anomalies. Note that we have focussed here specifically on the observed anomalies in the equatorial west Pacific, but this does not preclude a role for anomalies in the wider context of the Indo-Pacific warm pool. In particular, warm SST anomalies in the Indian Ocean have been shown to contribute to curbing the development of El Niño in 2014 (ref.^31^).

We also find that both forecast systems demonstrate a capability in simulating different levels of WWB activity in 2014 and 2015, with significantly more WWBs in 2015. This, and the clear relationship between WWB activity and subsequent ENSO development in forecasts, suggests the lack of WWB events in 2014 played a role in hindering El Niño development that year. WWBs are often thought to be caused through unpredictable internal variability in the atmosphere and as such may limit ENSO predictability^32^ but the WWB process clearly exhibits seasonal predictability in the cases examined here. There is a dependence on SST^33,34^ and May 2015 was generally warmer in the tropical Pacific than May 2014, with an equatorial (2°N-2°S) eastward extension of the warm pool of about 15° longitude. This seems a possible factor in explaining the difference in WWBs. Our analysis shows that in general the level of WWB activity in late spring is linked to the level of subsequent El Niño growth. For the two periods in 2014 and 2015 examined in this study, S4 has approximately double the mean WWBI of G5 and also larger variance. We speculate that this could be a contributing factor to the larger ensemble spread seen in SST plumes for S4 and the extreme positive SST anomalies that occur in some of the S4 ensemble members.

The skill of SST predictions at seasonal lead times is very high in the tropics and correlation scores for the ENSO peak season in winter are around 0.9 or more in modern forecast systems, including the G5 and S4 systems used here (e.g. refs^35–37^). For these and other systems, similar scores are attained for associated variables such as tropical precipitation^38,39^. Despite this success, closer examination of probabilistic reliability scores show that these forecasts are actually over-confident in the tropics and so forecast probabilities vary more than the corresponding observed frequency of occurrence for many tropical variables and thresholds (e.g. refs^40,41^). There are various possible explanations for this. Errors in the mean state can affect the development of anomalies. For example, a too shallow thermocline in the Eastern Pacific can result in the overestimation of the thermocline feedback, which will lead to stronger than observed anomalies^42^. In this case, a simple scaling of the variance of the resulting anomalies will produce more reliable forecasts. Another typical model error is the excessive cold-tongue erosion of the warm pool, which limits the occurrence of WWBs close to the dateline. In this case, adding stochastic WWB may help with the forecast reliability, but it would not address the underlying error in the coupled model. Forecast reliability may also be improved by better representation of noise-driven errors^8^ but developing parameterizations that enhance the reliability of the forecasts by better representation of the underlying physical mechanisms is the ultimate goal.

Here we have explored the 2014 case as an example of this overconfidence and the associated forecast bust in some systems, though we note that that the initialisation of these forecasts in May falls within the ‘spring predictability barrier’, a period when forecasts in general have relatively lower confidence^43^. Taken at face value, a probabilistic interpretation of the joint ensemble would be that the likelihood of the ENSO state remaining at ENSO-neutral/weak El Niño over 2014/15 was of low probability, highlighting the importance of making full use of probabilistic information. However, interesting differences have been identified between the two systems examined here, in particular with regard to their relative ability to maintain the cold southeast Pacific anomaly and with respect to simulation of WWBs. Errors in the climate models at the core of the ensemble prediction systems (or perhaps even from the initialisation) contribute to the lack of forecast quality and future studies should try to diagnose these errors, with a view to future improvement in forecast skill and reliability^44^.

## Methods

### Operational forecast systems in 2014 and 2015

The Met Office seasonal forecast system is GloSea5 (ref.^35^). The underpinning Met Office climate model used in the GloSea5 system was upgraded in March 2015. Forecasts for 2014 use the GA3.0 (ref.^35^), and forecasts for 2015 use the GC2 model^45^. In both cases the model resolution is the same, with a vertical resolution of 85 levels in the atmosphere (with a top at 85 km) and 75 levels in the ocean (with a 1 m top level). The ocean horizontal resolution is 0.25° on a tri-polar grid and in the atmosphere a horizontal resolution of 0.83° (longitude) x 0.56° (latitude) is used.

The ECMWF seasonal forecast system used in this study is System4 (ref.^46^). The atmosphere model is CY36R4 of ECMWF’s weather forecasting model Integrated Forecasting System. The resolution used in seasonal forecasting is TL255 with around 0.7° resolution for grid point calculations. The atmospheric model has 91 vertical levels reaching up to 0.01 hPa. The ocean model used in System 4 is NEMO version 3.0. The ocean model has 42 levels in the vertical, and the horizontal resolution is 1°, with enhanced latitudinal resolution in the tropics.

Forecasts for G5 are run daily with 2 members per day. Here the ‘May’ forecast refers to an ensemble consisting of 42 members constructed from 21 days preceding 1st May. Forecasts with System 4 are produced at the beginning of May using 51 ensemble members. For the purposes of making a comparison between the two systems we use the same years from the hindcasts for bias correction, 1996–2009 (14 years).

G5 and S4 operational forecasts for the period 2014 and 2015 are available from the Met Office and ECMWF web sites respectively:

http://www.metoffice.gov.uk/research/climate/seasonal-to-decadal/gpc-outlooks

http://www.ecmwf.int/en/forecasts/charts/catalogue

We note that operational products may differ from those presented here for a number of reasons. For example; monthly mean products on the Met Office web site for G5 use a different subset of days for constructing the forecast, S4 products on the ECMWF web site use a longer hindcast set for bias correction (30 years), and S4 SST plumes are scaled by variance.

#### WWB index

WWBs are identified here as events where the daily 10 m zonal wind anomaly (averaged from 2.5°N-2.5°S) exceeds 5 ms^−1^ over a longitude range of at least 10°, and lasts for at least 2 consecutive days in the equatorial Pacific (130°E- 80°W). We define a westerly wind burst index as the sum of the maximum anomaly for each event over a given time period, i.e. a measure of accumulated strength.

#### Verification data

Data for the forecast systems is compared with monthly mean SST from HadISST1.1 (ref.^47^) and daily 10 m winds and monthly mean 850hPa winds are from ERA-Interim reanalysis^48^. Anomalies for both datasets are calculated with respect to the respective 1996–2009 climatologies for consistency with the analysis of forecast anomalies.

#### Data availability

The SST observations data used in this study is available from the Met Office Hadley Centre via https://www.metoffice.gov.uk/hadobs/ and the ERA-Interim reanalysis data is available from https://www.ecmwf.int/en/forecasts/datasets/reanalysis-datasets/era-interim. The data used to produce the figures is available from the corresponding author for research use only.

## Electronic supplementary material

Supplementary Information
