# Supplementary material for: Predicting El Niño in 2014 and 2015
Source: Sci Rep. 2018 Jul 16;8:10733. doi: 10.1038/s41598-018-29130-1 (PMC6048137; doi:10.1038/s41598-018-29130-1)
Supplement: Supplementary file 1 — Supplementary Information [file 41598_2018_29130_MOESM1_ESM.pdf]

Supplementary Information for

Predicting El Niño in 2014 and 2015

S. Ineson[1*], M. A. Balmaseda[2], M. K. Davey[3], D. Decremer[2], N. J. Dunstone[1], M. Gordon[1], H. Ren[4,5], A. A. Scaife[1,6], and A. Weisheimer[2,7]

[1]Met Office Hadley Centre, Exeter, UK

[2]European Centre for Medium- Range Weather Forecasts, Reading, Berks, UK

[3]Department of Applied Mathematics and Theoretical Physics, University of Cambridge, Cambridge, UK

[4] Laboratory for Climate Studies & CMA-NJU Joint Laboratory for Climate Prediction Studies, National Climate Center, China Meteorological Administration, Beijing 100081, China

[5] Department of Atmospheric Science, School of Environmental Studies, China University of Geoscience, Wuhan, 430074, China

[6]College of Engineering, Mathematics and Physical Sciences, University of Exeter, Exeter, UK

[7]National Centre for Atmospheric Science (NCAS), Department of Atmospheric, Oceanic and Planetary Physics, University of Oxford, Oxford, UK

Atmospheric Bjerknes feedback

S4 exhibits more wind variability than G5. To further explore the different wind sensitivities found in these forecast systems the ENSO atmospheric dynamical feedback term, the Bjerknes feedback (e.g. Lloyd et al., 2009), is calculated for G5 and S4. This term acts as a positive feedback and is usually calculated as the regression of west Pacific low-level wind or wind stress anomaly on the east Pacific SST anomaly.

The regression of mean May-June 10m zonal wind anomaly on Niño3 SST anomaly for G5 and S4 hindcast data (1996-2009) is shown in Fig. S1a and S1b. The regression pattern is broadly similar for the two models, with the maximum variability shifted somewhat westward in S4. The regression coefficient for areas shown in Fig. S1 is 0.82 for G5 (1.08 for the G5-GA3.0 model used for the 2014 forecast/hindcast) and 0.89 for S4. Compared to observations, for which the regression coefficient is 1.49, the models show a weaker dynamical coupling between the ocean and atmosphere. This agrees with other analyses; in general this term is significantly underestimated by coupled models (Bellenger et al., 2014). However, the uncertainty for the observations is large for these 14 years, and, not surprisingly, the regression is sensitive to whether the extreme El Niño year of 1997 is included.

Given the similarity in patterns and magnitudes between G5 and S4 this linear analysis does not provide a clear reason for the differences in sensitivity between the two systems.

Impact of westerly wind bursts on El Niño development in May 2014 and May 2015 forecasts - removing the low frequency wind component.

Here we test the sensitivity of our results to removing the low frequency wind response to ENSO in the wind burst index calculation.

The monthly mean 10m zonal wind anomalies are regressed against Niño3 SST anomalies for all hindcast data resulting in spatial patterns for each month (similar to Fig. S1). The low frequency component of the wind response to ENSO in the forecasts is then approximated by multiplying these spatial patterns by the monthly mean forecast Niño3 SST anomaly for each ensemble member. The resulting monthly mean winds are smoothly interpolated to daily frequency and subtracted from the forecast 10m zonal wind anomaly.

The adjusted WWBI tends to be lower (Fig. S2) compared to the unadjusted WWBI (Fig. 6), as expected, but overall the results still hold. There are significantly more wind bursts in the first two months of the forecasts in 2015 than in 2014, implying a level of predictability in these years. In 2015 there appears to be a clear relationship between wind burst index and final forecast ENSO temperature (combined correlation of G5 and S4 is 0.51). S4 produces more westerly wind bursts than G5; in 2014 the mean WWBI for S4 is $7.2 ms^{-1}$, G5 is $5.6 ms^{-1}$ and in 2015 S4 is $43.7 ms^{-1}$ whereas G5 is $23.4 ms^{-1}$.

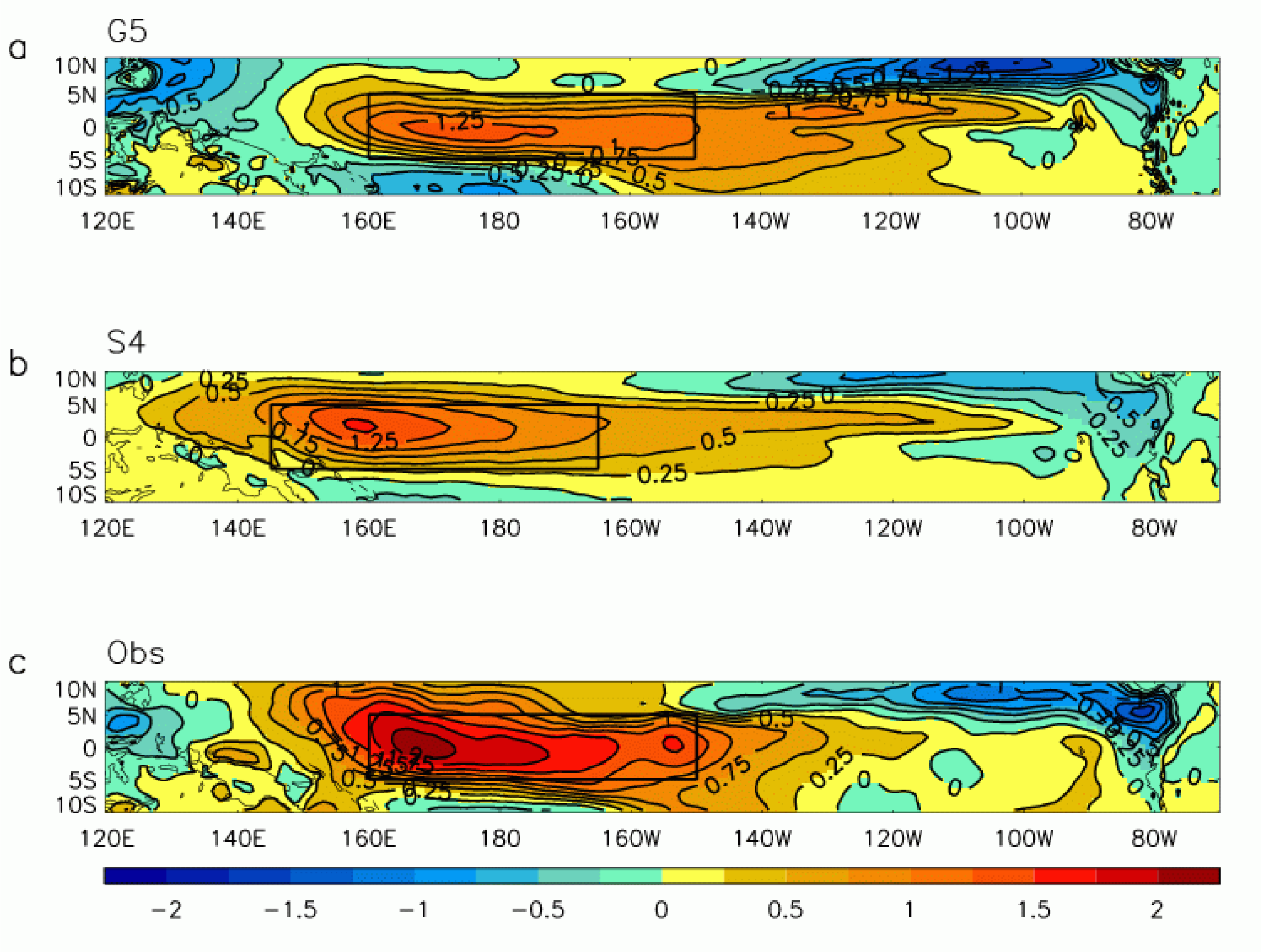

Supplementary Figure S1. Atmospheric Bjerknes feedback. Regression coefficient ($ms^{-1}K^{-1}$) of mean May-June 10m zonal wind anomaly on Niño3 SST index for 1996-2009 for a) G5, b) S4, and c) observations, 10m zonal wind from ERA-Interim and SST from HadISST1.1. The Niño4 region (160°E-150°W, 5°N-5°S) is shown in (a) and (c); the similar box in (b) maximises the regression for S4 and is located at (145°E-165°W, 5°N-5°S). Figure was created using IDL8.2 (http://www.harrisgeospatial.com/SoftwareTechnology/IDL.aspx), Met Office (in-house) release 8.2.0.10.

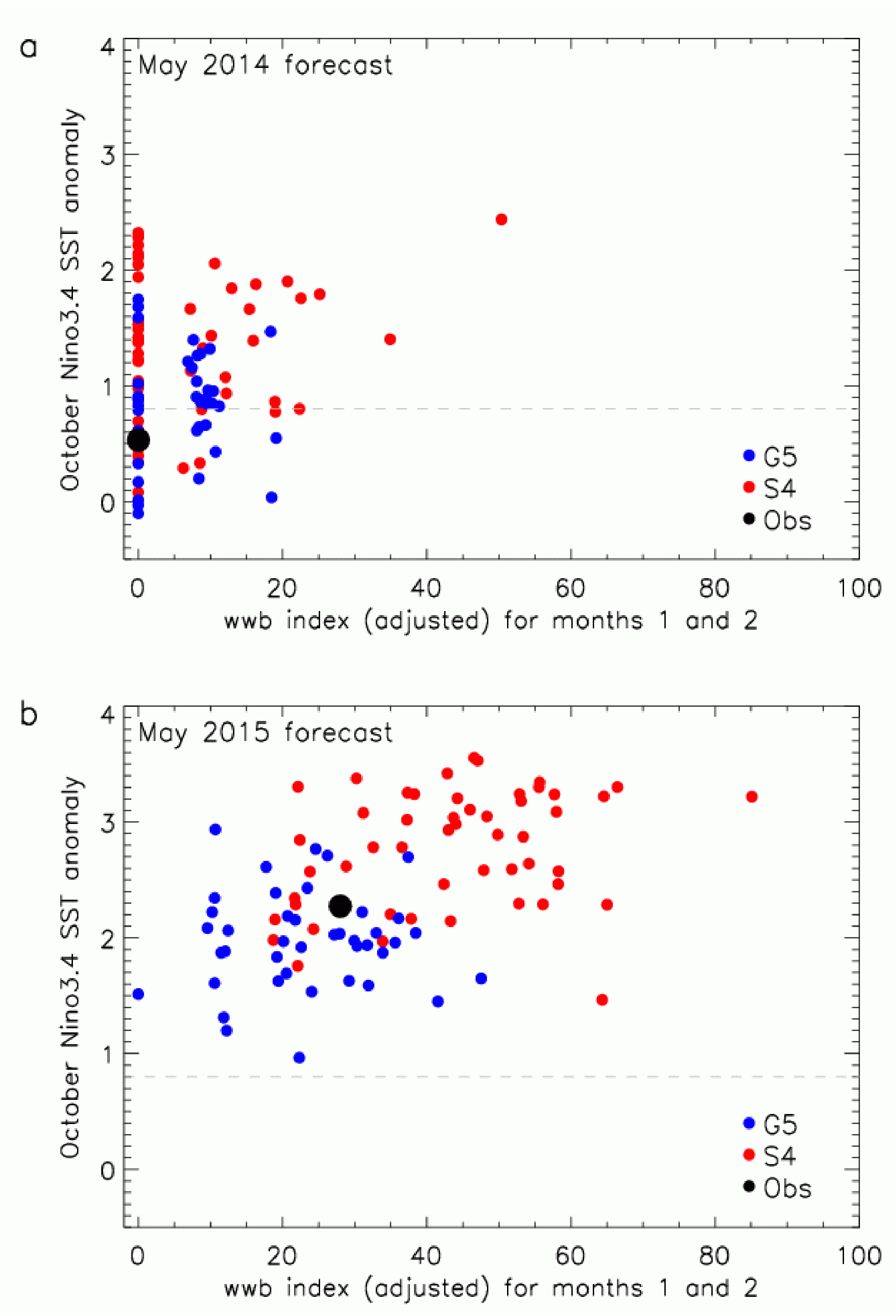

Supplementary Figure S2. The impact of westerly wind bursts on El Niño development in May 2014 and May 2015 forecasts. Scatter diagram of forecast ensemble members showing October Niño 3.4 SST anomaly against a westerly wind burst index (see Methods). Here, the low frequency wind variability associated with the Bjerknes feedback has been removed from the 10m wind anomaly. The index is for the first two months of the forecasts for G5 (blue circles) and S4 (red circles). Observations (black circles) are HadISST1.1 and ERA-Interim (u10 winds). The dashed grey line indicates the 0.8°C threshold.

References

Lloyd, J., Guilyardi, E., Weller, H., & Slingo, J. The role of atmosphere feedbacks during ENSO in the CMIP3 models. *Atmospheric Science Letters*, **10**, 170-176 (2009).

Bellenger, H., Guilyardi, E., Leloup, J., Lengaigne, M., & Vialard, J. ENSO representation in climate models: from CMIP3 to CMIP5. *Clim. Dyn.*, **42**, 1999-2018 (2014).
